# Assessing the prevalence of sensory and motor impairments in childhood in Bangladesh using key informants

**DOI:** 10.1136/archdischild-2014-305937

**Published:** 2014-07-08

**Authors:** Gudlavalleti V S Murthy, Islay Mactaggart, Muhit Mohammad, Johurul Islam, Christiane Noe, Aynul Islam Khan, Allen Foster

**Affiliations:** 1 Faculty of Infectious & Tropical Diseases, Department of Clinical Research, International Centre for Evidence in Disability, London School of Hygiene & Tropical Medicine, London, UK; 2 Child Sight Foundation, Dhaka, Bangladesh; 3 Knowledge Learning & Training Department, CBM International, Bensheim, Germany; 4 Department of Paediatric Nephrology, National Institute of Kidney Disease and Urology, Dhaka, Bangladesh

**Keywords:** Comm Child Health, Deafness, Musculo-Skeletal, Tropical Paediatrics

## Abstract

**Objectives:**

The study was conducted to determine whether trained key informants (KI) could identify children with impairments.

**Design:**

Trained KI identified children with defined impairments/epilepsy who were then examined by a medical team at a nearby assessment centre (Key Informant Methodology: KIM). A population-based household randomised sample survey was also conducted for comparing the prevalence estimates.

**Setting:**

Three districts in North Bangladesh.

**Participants:**

Study population of approximately 258 000 children aged 0–<18 years, within which 3910 children were identified by KI, 94.8% of whom attended assessment camps. In the household survey, 8120 children were examined, of whom 119 were identified with an impairment/epilepsy.

**Main outcome measures:**

Prevalence estimates of severe visual impairment (SVI), moderate/severe hearing impairment (HI), substantial physical impairment (PI) and epilepsy.

**Results:**

Overall prevalence estimates of impairments, including presumed HI, showed significant differences comparing KIM (9.0/1000 (95% CI 8.7 to 9.4)) with the household survey (14.7/1000 (95% CI 12.0 to 17.3)). Good agreement was observed for SVI (KIM 0.7/1000 children: survey 0.5/1000), PI (KIM 6.2/1000 children: survey 8.0/1000) and epilepsy (KIM 1.5/1000 children: survey 2.2/1000). Prevalence estimates for HI were much lower using KIM (2/1000) compared to the survey (6.4/1000). Excluding HI, overall prevalence estimates were similar (KIM: 7.5/1000 children (95% CI 7.2 to 7.8) survey: 8.4/1000 (95% CI 6.4 to 10.4)).

**Conclusions:**

KIM offers a low cost and relatively rapid way to identify children with SVI, PI and epilepsy in Bangladesh. HI is underestimated using KIM, requiring further research.

**What is already known on this topic**

- There are huge gaps in evidence to plan health services for children with disability in low/ middle-income countries.
- Key informants have been used to identify children with epilepsy and blindness

**What this study adds**

- Key informants can be trained to identify children with severe visual impairment, substantial physical impairment and epilepsy.
- Key informants offer a low-cost relatively rapid method to identify children with disability in low/middle income countries.

## Introduction

The lack of data on the prevalence and causes of functional impairment in children in low income countries (LMIC) is a major barrier to providing appropriate health services to meet their needs.^1^ WHO reports a prevalence of ‘severe disability’ among children as 7/1000 (0–14 years),^1^ with an estimated 93 million children worldwide in need of services related to their disabilities.^2^

Impairment refers to an individual's functional capacity as influenced by health conditions or trauma. Disability refers to the interaction between an individual's functional capacity and environmental and personal factors.

Planning for health services for children at national and district levels requires quality information on the numbers of children affected by functional impairment. Furthermore, these numbers are of benefit to other service providers working with children with disabilities, including inclusive education and community-based rehabilitation.

Key informants (KI) are persons whose professional or organisational role at the community level allows them to influence community decision making.^3^ KI have previously been used to identify epilepsy,^4^ mental health needs,^5^ maternal mortality^6^ and childhood blindness.^7^^–^11

## Materials and methods

A pilot study was conducted to determine the ability of KI to identify children with different impairments. Five subdistricts of Sirajganj district in North Bangladesh were randomly selected and KI were trained to exclusively identify children with severe visual impairment (SVI), moderate/severe hearing impairment (HI), physical impairment (PI) and epilepsy, in four subdistricts, respectively. In the fifth subdistrict, KI were trained to identify all the above impairments. Children listed by KI were examined by a medical expert at an assessment camp. The pilot showed that training KI to identify SVI, HI, PI and epilepsy simultaneously was more acceptable and efficient than identifying each impairment/epilepsy separately (sensitivity: 99%; specificity: 24%). These results have been reported previously.^12^

### Study area and population

The study area consisted of 11 rural subdistricts (upazillas) randomly selected from three districts (Sirajganj, Natore and Bogra) in the Rajshahi Division of Bangladesh, with a total all-age population of 600 000 and a study population (age 0–<18 years) estimated at 258 000.

### Key informant methodology

The study used a two-stage process of identification and assessment of children with impairments/epilepsy which we refer to as the key informant methodology (KIM). KIM is, therefore, a combination of identification and listing of children by the KI at the village level, and examination by medical specialists at a clinical assessment camp.

### Identification of children with impairments by KI

KI were trained to identify children with SVI, moderate/severe HI, substantial PI and epilepsy, according to standardised case definitions. Intellectual impairment was not included.

KI included workers from village-level government/non-government agencies, school teachers, elected community leaders, religious leaders (imams) and health workers. They were given a 1-day training in Bengali, (groups of 20–25) on finding children with impairments/epilepsy using previously tested flip books, and written information with visual illustrations, and a list of key features required for case finding.

The KI training was conducted by four trained salaried supervisors with prior experience in conducting a KIM for childhood blindness in Bangladesh.^7^ Supervisors undertook an intensive 4-week training, including identification of children with impairments, and sensitisation to disability issues.

After training, the KI motivated their communities to provide the names of children with suspected impairments within a 5-week window. KI made announcements and coordinated discussions at prayer meetings, market places, self-help group meetings, and other community events. Each child listed by the KI was visited by the supervisors to confirm the child's name and invited the parents and child to attend a medical assessment camp.

### Assessment camp

The assessment camps were held in villages within 10 km of the child's home. The assessment team included an ophthalmologist, ear, nose, throat (ENT) specialist, audiologist, paediatrician, physiotherapist, community disability worker, and counsellor who performed examinations, made provisional diagnoses, provided advice, and referred children to appropriate services and treatments. Assessment included visual acuity (VA) testing, otoacoustic emission (OAE) and pure tone audiometry (PTA) testing, and a physical examination. A medical record was given to the caregivers of each child requiring a referral, and this was recorded in a register for follow-up support, with initial referral costs subsidised by the project. Full assessment lasted approximately 45–60 min, with between 100 and 200 children attending each camp. Transport was arranged for those otherwise unable to attend.

### Population-based survey

A population-based survey was also conducted to determine prevalence of impairments in the same districts for comparison with the KIM prevalence estimates. A sample size of 8900 children aged 0–<18 years was calculated using a prevalence of 16/1000 95% CIs, 25% relative error, design effect of 2.0 and a response rate of 85%. Cluster random sampling using population proportionate to size principles led to random selection of 45 clusters each of approximately 200 children aged 0–<18 years individuals. The survey was conducted at the same time as the assessment camps, using the same case definitions. All children received OAE testing and VA examination by a trained paramedical worker, followed by examination by a paediatrician.

### Definitions used

A child was defined as a person below 18 years of age (0–<18).^13^

PI was defined as ‘substantial’ impairment of 6 months duration (or from birth), affecting functions as per the Washington Group Questions on functional limitations in core domains.^14^

SVI was defined as presenting vision of <6/60 in the better eye or inability to follow light (if age <5 years).^7^

Moderate/severe HI was defined as presenting hearing loss of >30 decibel level A weighted (dBA) in ears,^15^ or failure of OAE test in both ears (if age <5 years). If the ear was discharging (preventing PTA/OAE test), a strong clinical suspicion of HI on examination by an ear specialist was accepted.

Epilepsy was defined by a history of generalised tonic-clonic seizures of more than 3 months, with at least two episodes in the 3-month period.

### Data management and analysis

Data was entered into a purpose-developed access software package. All forms were checked for completeness first in the field and second at the project office in Dhaka. Double data entry was performed and inconsistencies resolved. Data cleaning was completed at the Indian Institute of Public Health, Hyderabad, and analysis was done using Stata V.12.0 at the London School of Hygiene and Tropical Medicine (LSHTM), London.

### Informed consent

Informed consent was obtained from the parents of all children participating in the study. The study purpose was explained in the local language, and a signature or thumb impression (illiterate individuals) was obtained for those willing to participate. People who refused to participate were not discriminated in any manner. Basic medical services were provided to all children requiring them, irrespective of their participation. Strong links were established with local and national specialist referral centres and community-based rehabilitation (CBR) programmes so that children requiring specialist care could receive necessary treatment.

## Results

### Profile of key informants

One thousand five hundred and ten KI including persons with disabilities were trained. Each KI identified an average of 2.6 children to attend the assessment camps.

### Identified children

Of the 3910 children identified by KI, 3707 (94.8%) attended the camps. The attendance rate was greater than 90% across all age groups, although this decreased with increasing age (X_4_^2^-63.41; p<0.001). (table 1)

**Table 1:** Age and gender of children identified by key informants (KI) and attending screening camps

Age (years)	Children listed by KI						Children examined by medical team at assessment camps								
	Male		Female		Total		Male			Female			Total		
	n	Per cent	n	Per cent	n	Per cent	n	Per cent	Attendance rate %	n	Per cent	Attendance rate %	n	Per cent	Attendance rate %
≤5	589	26.9	440	25.5	1029	26.3	587	28.2	99.7	431	26.5	97.9	1018	27.5	98.9
6–10	774	35.4	573	33.3	1347	34.4	732	35.1	94.6	538	33.1	93.9	1270	34.3	94.3
11–15	479	21.9	452	26.3	931	23.8	464	22.3	96.9	411	25.3	90.9	875	23.6	94.0
16–18	346	15.8	257	14.9	603	15.4	301	14.4	87.0	243	15.0	94.5	544	14.7	90.2
Total	2188	56.0	1722	44.0	3910	100	2084	56.2	95.2	1623	43.8	94.2	3707		94.8

### Age and gender distribution of referrals

Among those listed by KI, 56% (2188) were male, 26% were aged 0–5 years, 34% 6–10 years, 24% 11–15 years and 15% 16–<18 years. The age and gender distributions of those attending the assessment camps were similar.

More male than female children were referred by KI and attended the assessment camps in all age groups (table 1).

Among the 3707 children assessed at the camps, 63% (2334) had an impairment/epilepsy as defined by the study. A further 634 (17%) had unilateral or other impairments not meeting the case definitions of the study, and 553 (15%) had chronic or acute medical conditions. Only 186 (5%) children attending the assessment camps did not have any impairment/epilepsy warranting a clinical examination (table 2).

**Table 2:** Clinical findings of children listed by key informants (KI) who were examined at assessment camps

Characteristics	Number	Per cent
Children listed by KI with suspected impairment	3910	100
Children listed by KI attending assessment camps	3707	94.8
Children assessed as having an impairment as per study definitions	2334	63.0
Children listed by KI not having severe impairment as per study criteria, but having unilateral or other non-targeted impairments	634	17.1
Children listed by KI found to have other chronic health conditions	241	6.5
Children listed by KI found to have an acute illness	312	8.4
Children listed by KI, but found to have no health problem	186	5.0

### Prevalence of impairments in children

Based on census data,^16^ the subdistricts included in the study had an estimated population of 258 000 children aged 0–<18 years, giving an estimated prevalence of childhood impairments using KIM of 9/1000 (95% CI 8.7 to 9.4) (table 3).

**Table 3:** Comparison of prevalence estimates by impairment/health condition using key informants (KI) method and population-based household survey

Impairments/health conditions	KIM camps (258 000) (<18 years population covered)			Household survey ( 8120) (<18 years population covered)		
	n	Per 1000	95% CI	n	Per 1000	95% CI
All children identified as having an impairment	2334	9.0	8.7 to 9.4	119	14.7	12.0 to17.3
All children identified as having an impairment excluding those with hearing impairment	1937	7.5	7.2 to 7.8	68	8.4	6.4 to 10.4
By specific health condition/impairment*						
Generalised seizure disorder	390	1.5	1.4 to 1.7	18	2.2	1.2 to 3.2
Physical impairment	1601	6.2	5.9 to 6.5	65	8.0	6.1 to 9.9
Bilateral visual impairment	184	0.7	0.6 to 0.8	4	0.5	0.01 to 1.0
Bilateral hearing impairment (Audiometry)	86	0.3	0.2 to 0.4	52	6.4	4.7 to 8.1
Bilateral hearing impairment (Presumed based on clinical diagnosis or audiometry)	513	2.0	1.8 to 2.2			

*Some children have a physical and/or visual and/or hearing impairment, so that the individual prevalence figures do not add up to the overall prevalence figures (312 children in KIM had more than one impairment, that is, 13.4% of all children with an impairment).

KIM, key informant methodology.

The prevalence of childhood impairments observed in the survey was 14.7/1000 children (95% CI 12.0 to 17.3) (table 3).

There was good agreement in the prevalence results between KIM and the survey for PI (KIM 6.2/1000 children: survey 8.0/1000), SVI (KIM 0.7/1000 children: survey 0.5/1000), and epilepsy (KIM 1.5/1000 children: survey 2.2/1000). However, the prevalence for HI was much lower using KIM compared to the survey (2.0/1000; KIM: 6.4/1000 survey) (table 3).

**Figure 1:**
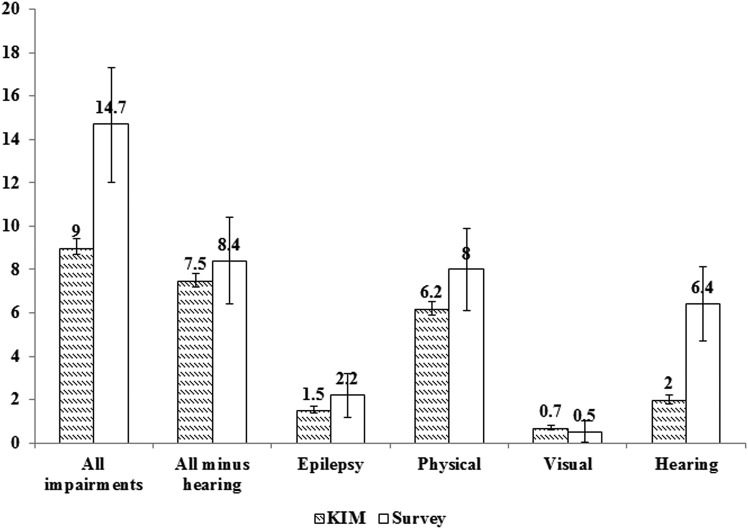
Comparison of prevalence estimates from key informant methodology and population-based house-to-house survey.

The overall prevalence estimate of the impairments/epilepsy, as per study definitions (excluding HI) was 7.5 per 1000 children (95% CI 7.2 to 7.8) in the KIM compared to 8.4 per 1000 (95% CI 6.4 to 10.4) from the survey. However, the overall prevalence of impairments, including HI, showed a significant difference between the two methods (KIM—9.0/1000 KIM (95% CI 8.7 to 9.4) vs survey—14.7/1000 (95% CI 12.0 to 17.3)) (figure 1).

### Types of impairments

Of the children with impairments referred by KI, PI was found in 69% (1601/2334), epilepsy in 17% (390/2334), and SVI in 8% (184/2334). In the survey, PI was found in 55% (68/119); HI in 44% (52/119), epilepsy in 15% (18/119) and SVI in 3% (4/119). Some children had multiple impairments/epilepsy.

Cerebral palsy was the predominant cause of PI in KIM (59.5%) and the survey (32.8%). Other causes of PI included club foot (9% in KIM; 3.1% in survey), cleft lip/cleft palate (6.7% in KIM; 1.6% in survey), trauma/burn (8.4% in KIM; 15.6% in survey) and muscular dystrophy (7.5% in KIM; 1.6% in survey).

### Cost of KIM and population-based survey

The costs incurred in conducting the KIM and the population-based survey was calculated. The total cost for KIM to cover a population of approximately 258 000 children and identify 2334 children with impairment was estimated at £210 000 giving a unit cost of £90 per child with an impairment identified. The costs to conduct the survey were estimated at £42 000, from which 119 children with impairment were identified (equating to £352 per child). The gross domestic product per capita expenditure on health in Bangladesh is estimated at £16 per annum.

## Discussion

### Study limitations

The study showed that the identification of PI, epilepsy and SVI using the KIM was a useful technique, but the method was not effective for HI. This study did not include children with intellectual impairment or target children with mild or unilateral impairments, but focussed on children with significant motor and sensory impairments or epilepsy. We found that the KIM underestimated the prevalence of HI compared with the survey. This may be due to the difficulty in identifying children with HI, as hearing loss is not something which is visible to the naked eye. Additionally, confirming the presence of HI at the assessment camp was challenging given the high number of children presenting with discharging ears (OAE and PTA tests not possible). Further work is therefore required on the training of KI to identify children with hearing loss, and on appropriate tests to confirm a child as having a HI in a field setting. Though the prevalence estimates for SVI were similar using KIM and the survey, the 95% CI observed in the survey (0.01 to 1.0) is wide, and the observation could have occurred by chance. Since the prevalence of SVI is very low in these communities, a much larger sample would have been required for SVI. The KIM seems a better option to identify these children in resource-poor settings at a much lower cost, compared to a survey.

The comparison of KIM with the population-based survey is valid for the prevalence estimates at the group level, but the study did not focus on test characteristics (sensitivity/specificity) at individual level.

### Gender differences

There may be several reasons for the gender differences observed in the study. Boys may have a greater incidence of impairment due to sex-linked conditions, while girls with impairments may have a higher mortality compared to boys. There may also be more stigma associated with functional impairment in girls compared with boys.

### Ascertaining the prevalence of impairments in children

Prevalence estimates for childhood impairments vary greatly across low and middle income countries (LMIC) from approximately 1–45%.^2^ ^17^^–^27 This wide range of estimates is due to the use of different definitions, age groups and measurement tools. The 10-item parent-reported questionnaire has been the commonest tool used to estimate child disabilities in most LMICs.^24^ Population-based surveys and census data can also provide prevalence estimates. However, due to low disability prevalence, surveys must be large (and therefore costly), while census data is limited by the questions that can be asked and subjective responses. The KIM provides a viable alternative to these methods in the identification of children with targeted clinical impairments and health conditions.

### Key informants

The use of KI to identify children with health conditions has been used successfully for childhood blindness and epilepsy.^4^ ^7^^–^11 20 26 KI are resident in the communities and, therefore, interact with the families on a regular basis, increasing trust levels^27^ and providing an opportunity for follow-up and continuity. The present study showed that the prevalence estimates of children identified by KI with PI, SVI and epilepsy was similar to the prevalence found in a survey, leading to the assumption that KI can effectively identify children with impairments.

KI offer a low-cost and relatively rapid way to reach rural communities in order to identify children with impairments who may benefit from health interventions. They can also provide a community-centred approach to improve the access to health services for children with disabilities.

### Planning impairment-specific health services

One of the objectives of the study was to obtain data to plan health services for children with impairments. In Bangladesh, disability estimates vary from 0.5% to 14% depending on the definitions used and the populations covered.^28^ A million total population is a useful denominator for planning health services as it approximates to the size of an administrative health unit and is user-friendly, facilitating advocacy and planning. The prevalence estimates from the KIM study were extrapolated to a million population (table 4) to prioritise service needs. The results indicate that in rural Bangladesh, approximately 2500 children per million total population have PI of which 1500 have cerebral palsy, 230 have club foot and 170 have cleft lip. A further 600 children per million total population have epilepsy, 300 SVI, and potentially 700 HI. These estimates based on the KIM study provide data for prioritising and planning the use of limited resources to meet children's health needs.

**Table 4:** Using the key informants (KI) Method to plan services for children with impairments per million total population

Condition	Number identified	Prevalence/1000 children		Number per million total population	
	n	Prevalence	95% CI	n/million	95% CI
Physical impairment affecting function	1601	6.2	5.9 to 6.5	2563	2438 to 2688
Cerebral palsy	953	3.7	3.5 to 3.9	1526	1429 to 1622
Bilateral hearing impairment (suspected clinically)	513	2.0	1.8 to 2.2	821	750 to 892
Generalised seizure disorder	390	1.5	1.4 to 1.7	624	562 to 686
Bilateral visual impairment	184	0.7	0.6 to 0.8	295	252 to 337
Club foot	144	0.6	0.5 to 0.7	231	203 to 280
Cleft lip	107	0.4	0.3 to 0.5	171	145 to 211

## Conclusion

In the present study, KI accurately identified children with PI, SVI and epilepsy, but underestimated those with HI. The KIM was able to identify and to provide referrals for children with impairments in need of health services in a large population over a relatively short period of time and at low cost. The data obtained from KIM can be used to prioritise and plan the allocation of resources for health services for children with impairment. Further work is required to develop tools to include children with intellectual impairments and to improve the diagnostic accuracy for testing hearing of children at community level.
